# Measuring Health Literacy Among Adults with HIV Infection in Mozambique: Development and Validation of the HIV Literacy Test

**DOI:** 10.1007/s10461-016-1348-3

**Published:** 2016-03-10

**Authors:** José A. Tique, Leigh M. Howard, Sandra Gaveta, Mohsin Sidat, Russell L. Rothman, Sten H. Vermund, Philip J. Ciampa

**Affiliations:** 1 National STI’s and HIV Program, Ministry of Health, Avenida Eduardo Mondlane/Salvador Allende, Maputo, Mozambique; 2 Department of Pediatrics, Vanderbilt University School of Medicine, Nashville, USA; 3 Community Health Department, Eduardo Mondlane University, Maputo, Mozambique; 4 Departments of Pediatrics and Medicine, Vanderbilt University School of Medicine, Nashville, USA; 5 Harvard Medical School, Harvard University, Boston, USA

**Keywords:** Health literacy, Mozambique, HIV, Health communications, Antiretroviral therapy, Psychometrics, Poverty

## Abstract

The role of health literacy on HIV outcomes has not been evaluated widely in Africa, in part because few appropriate literacy measures exist. We developed a 16-item scale, the HIV Literacy Test (HIV-LT) to assess literacy-related tasks needed to participate in HIV care. Items were scored as correct or incorrect; higher scores indicated higher literacy skill (range 0–100 %). We tested internal reliability (Kuder–Richardson coefficient) of the HIV-LT in a convenience sample of 319 Portuguese-speaking, HIV infected adults on antiretroviral treatment in Maputo, Mozambique. Construct validity was assessed by a hypothetical model developed a priori. The HIV-LT was reliable and valid to measure participants’ literacy skills. The mean HIV-LT score was 42 %; literacy skills applicable to HIV care were challenging for many participants. The HIV-LT could be used to assess the relationship of literacy and HIV-related outcomes in diverse settings, and evaluate interventions to improve health communication for those in HIV care.

**Electronic supplementary material:**

The online version of this article (doi:10.1007/s10461-016-1348-3) contains supplementary material, which is available to authorized users.

## Introduction

Through substantial international efforts, there has been an unprecedented increase in access to HIV treatment in sub-Saharan Africa, where 70 % of the world’s 35 million HIV infected people reside [1]. Despite significant advances in antiretroviral therapy (ART) coverage, many patients still experience significant mortality after treatment initiation [2], and suboptimal adherence remains an important threat for opportunistic infections and HIV disease progression [3, 4]. While numerous barriers to patients’ adherence and retention to care in sub-Saharan Africa have been described [5–7], the role of health literacy on HIV related behaviors and outcomes has been a relatively neglected research topic.

Overall literacy skills include an individual’s ability to read, write and comprehend written language (print literacy), speak and understand spoken language (oral literacy) and understand and use numbers in daily life (numeracy) [8, 9]. Health literacy, a subset of overall literacy skills that is highly correlated with it, has been defined as “the degree to which individuals have the capacity to obtain, process and understand basic health information and services needed to make appropriate health decisions” [10]. Low or inadequate health literacy is common even in high-income countries such as the United States, where 90 million people are estimated to be affected [8]. Studies have shown that limited health literacy is associated with suboptimal health outcomes, particularly in chronic conditions such as diabetes and HIV [8, 11–13]. Despite the high levels of illiteracy found in sub-Saharan Africa, affecting up to 40 % of its population [14], studies examining the association between literacy or health literacy and health outcomes have been limited in number, scope and spread [15–20].

Successfully managing HIV infection requires from individuals the ability to access medical care, understand medical recommendations and execute treatment plans; these skills may each be influenced by an individual’s health literacy [8]. Evidence from the US has shown that health literacy skills are important mediators of HIV-related knowledge, behaviors and outcomes [21–28]. Studies suggest that individuals with HIV who have limited health literacy have less HIV knowledge [21–24], less ability to correctly manage HIV medication [25, 29], and lower likelihood of achieving undetectable viral loads compared to those with adequate health literacy skills [21, 27]. Most studies have demonstrated an association between limited health literacy and lower adherence to ART [26, 27, 30–33], but some did not find such a relationship [23, 34, 35].

In the few known studies examining the relationship between health literacy and HIV related behaviors and outcomes in sub-Saharan Africa, population literacy data and overall literacy skills have been used as surrogates for health literacy [19, 20]. While these types of data may not require specialized testing, they do not measure the specific literacy skills needed to participate in health-related activities. This moves away from the definition of health literacy that relates to an individual’s possession of requisite skills for making health-related decisions, meaning that health literacy must always be examined in the context of the specific tasks that need to be accomplished [51]. This underscores the need for measures that are applied to a disease or health context.

In Mozambique, where the 2009 prevalence of HIV among adults was 11.5 % [36], census data report that only 55 % of the population were literate in 2007 [14]. We do not know how such profound levels of limited literacy skills may impact the ability of adults to self-manage diseases like HIV infection in low-income settings such as Mozambique, or what impact limited health literacy may have on health behaviors and outcomes. The objective of this study was to develop and rigorously test the psychometric properties of a novel measure of health literacy for adults with HIV, the HIV Literacy Test (HIV-LT).

## Methods

### Scale Development

We developed the 16-item HIV-LT to assess common literacy and numeracy-related tasks that adults with HIV infection in Mozambique must perform in order to participate in HIV-related care. These tasks include the ability to dose oral medications, manage appointments, and understand the risk of HIV transmission and of treatment side effects. Key literacy and numeracy skills tested in the HIV-LT include document/print literacy, addition, multiplication, division, numeration/numerical hierarchy, fractions, and percentages. Preliminary work measuring general literacy and numeracy [19] and review of existing measures of health literacy applied to other diseases informed item development [37–39]. In the initial development phase, a pool of 35 items was generated. Scale items included clinical materials (such as prescription cards) used during routine HIV care in Mozambique whenever appropriate. Items were generated in English and translated into Portuguese by a native speaker and official translator, then back-translated to ensure content fidelity [40, 41]. To limit content redundancy, our panel of experts reduced this initial pool to 16 items that had high face validity to assess a range of literacy skills needed to participate in HIV care.

**Fig. 1:**
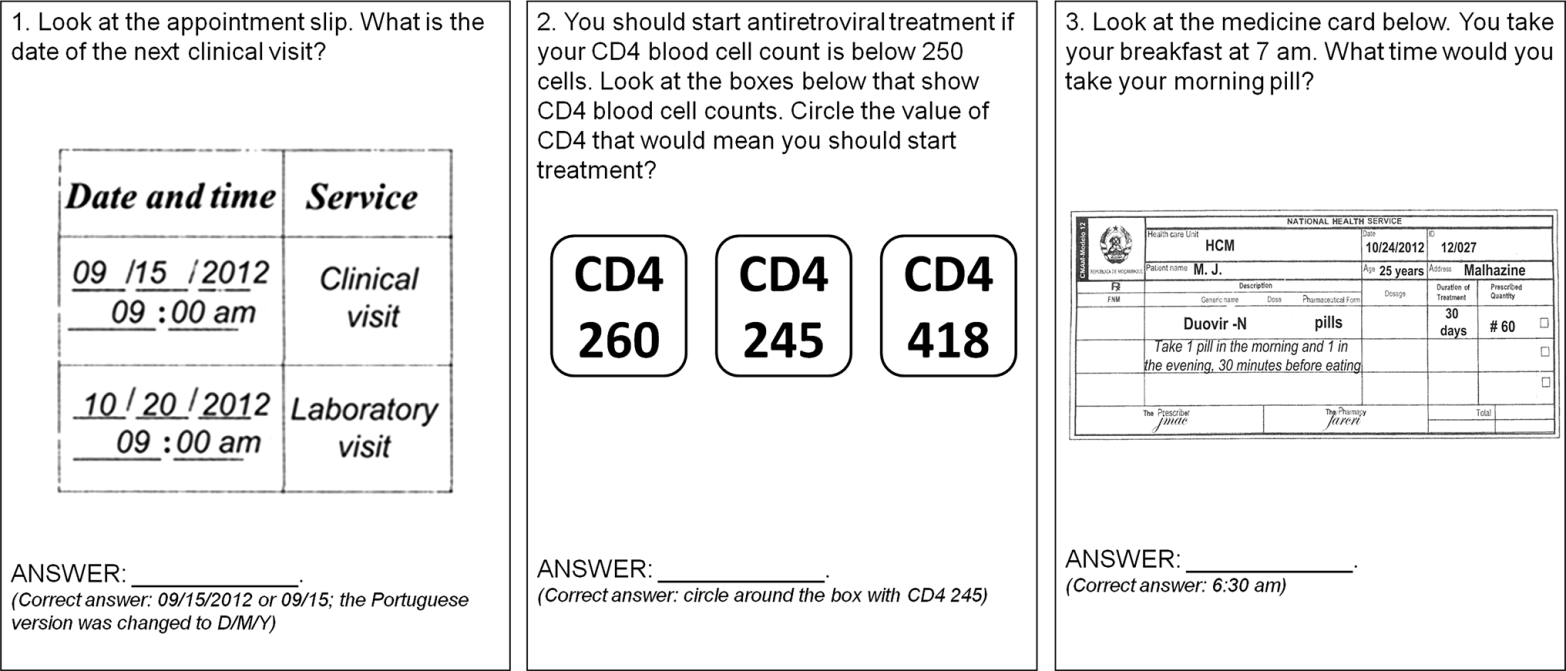
Sample items from the HIV Literacy Test (HIV-LT)

In the second phase of development, we conducted cognitive-based assessments with 20 Portuguese-speaking adults accessing HIV care and receiving ART at two clinics in Maputo, Mozambique to get feedback about the 16 candidate items. Participants with a level of formal education that ranged from none to 12 years were selected for the assessment. Participants were asked to evaluate the content, clarity, and readability of each item. Suggestions to simplify some of the medical terms used in the items and to apply culturally appropriate language were incorporated into the revision of scale items used in the study. No items were abandoned in this process. In order to ascertain health literacy and numeracy even for those participants with very limited literacy and numeracy, and assess participant’s skills in a format that is commonly used in usual practice, the first five item stems in the HIV-LT were read out loud for the participants. The rest of the items in the scale were self-administered. Item responses are defined as correct or incorrect with higher scores indicating higher literacy skill (range 0–100 %). Since there is no established minimum or maximum HIV treatment literacy threshold in the literature (and would likely vary by disease and local context), we did not define these score values in the HIV-LT. There was no time limit for responding to the questions (sample HIV-LT questions in Fig. 1).

**Fig. 2:**
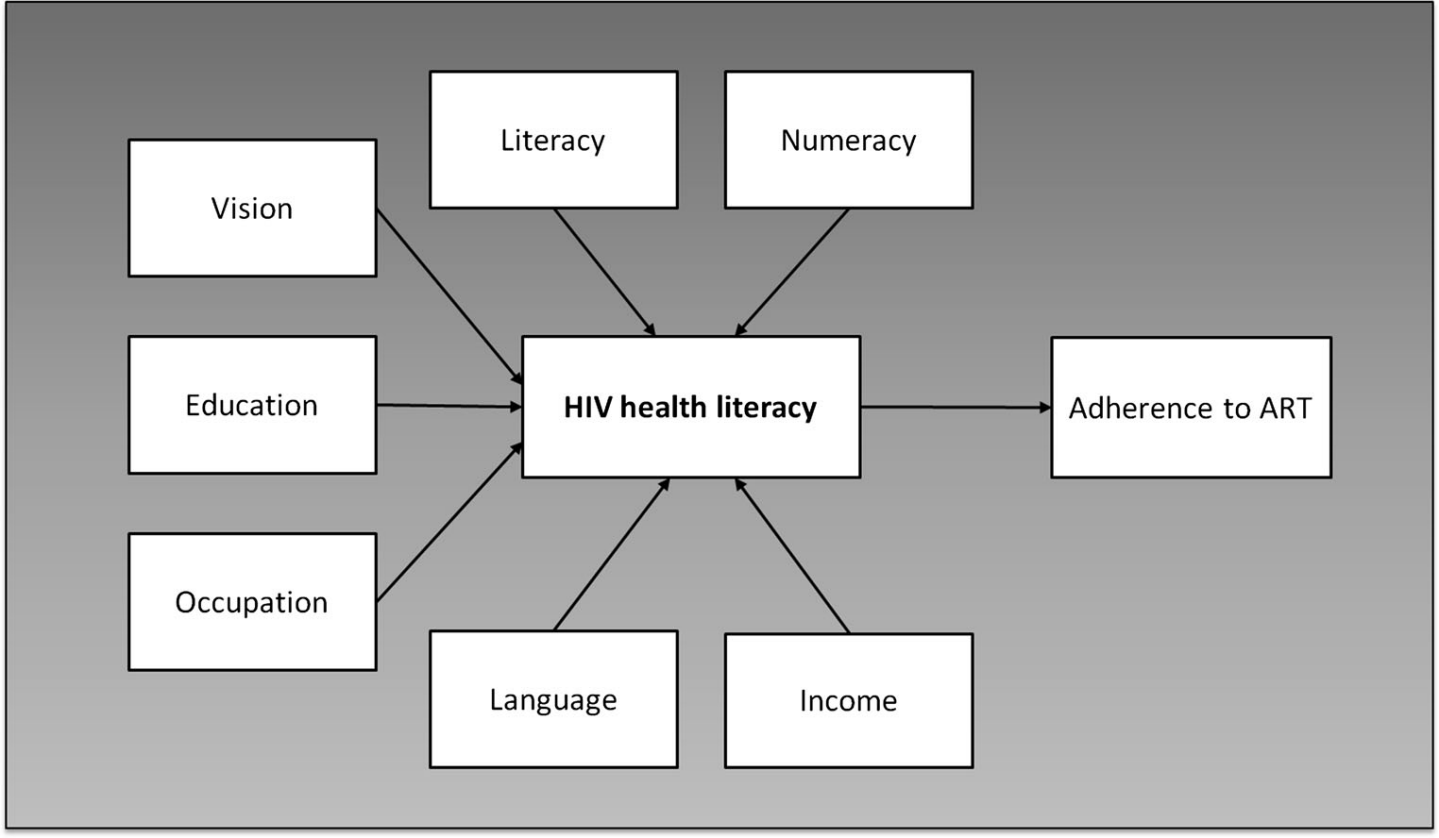
Model depicting constructs used to establish validity of the HIV Literacy Test

The third phase of development sought to assess the internal reliability and construct validity of the HIV-LT in a cross-section of adults on treatment for HIV infection in Maputo, Mozambique. Since there is no gold standard for HIV health literacy and numeracy, we adapted and used a model containing factors that would be expected to associate with health literacy to evaluate construct validity of the HIV-LT. Seven factors were selected a priori to test construct validity within this hypothetical model (Fig. 2). We hypothesized that higher HIV-LT scores would correlate with higher general literacy and numeracy skill, more education, higher income, work outside the home, and use of Portuguese as the primary language spoken at home. We also hypothesized that higher HIV-LT scores would be associated with higher levels of adherence to ART.

In the final phase of scale development, we used a split sample analysis to identify a shortened, more clinically useful 10-item version of the HIV-LT, the HIV-LT10. The data were randomly split into two smaller sub-samples, and we applied principal component factor analysis in sub-sample 1 to determine which items to include in the HIV-LT10. Items with <0.5 loading on the primary component, and those that had >80 % or <10 % of participants answering correctly were discarded. Two items that had been deemed a priori to have high content validity were added back, bringing the total number of items to 10. We used the sub-sample 2 to confirm the internal reliability and construct validity of the HIV-LT10. Internal reliability of the HIV-LT10 was evaluated by the Kuder–Richardson coefficient (KR-20), and its construct validity was assessed by establishing correlations with the original 16-item scale and the seven variables selected a priori to test construct validity.

### Study Design, Setting and Participants

We used a cross-sectional study design to test the psychometric properties of the HIV-LT. From August to November 2012, we recruited a convenience sample of 319 adults from two clinics that provide HIV-related care: (1) the Polana Caniço health center, an outpatient care facility in the city of Maputo, Mozambique, and (2) the Marracuene health center, 40 km away from Maputo, in a district with a more rural population. Two study investigators who are native Portuguese-speakers (JT, SG) were responsible for participant recruitment and survey administration. Participants were included if they were between 18 and 49 years of age, had an established diagnosis of HIV infection, and had been on ART for at least 3 months. Participants were excluded from the study if they were unable to communicate in basic Portuguese, and because the HIV-LT requires some reading and writing ability, participants that demonstrated a visual acuity of worse than 20/50 (if uncorrected) as determined with a pocket vision screener (Rosenbaum, Graham-Field Surgical Co, Inc, New Hyde Park, NY, USA) were also excluded.

### Measures

#### Demographics

Socio-demographic information was collected by structured interview and included age, primary language spoken at home, distance from home to the clinic, education, occupation, and income. HIV disease stage information was extracted from clinical records.

#### General Literacy and Numeracy

General literacy and numeracy were measured with a Portuguese-language adaptation of the Wide Range Achievement Test, version 3 (WRAT-3) word reading and numeracy sub-scales [42] that have been previously validated for use in Mozambican women [19]. The word reading subscale consists of a list of 15 letters and 42 words of escalating contextual and phonetic complexity and measures general literacy skill. Participants attempt to read each item on the list and are scored for proper pronunciation; scores range from 0 to 57. The arithmetic subscale consists of two parts, and measures general numeracy skill (scores range from 0 to 30). Part one consists of 15 orally administered items augmented by a visual aid card. A perfect score of 15 correctly answered items corresponded to the US equivalent of kindergarten or less skills. Part two consists of 15 self-administered arithmetic problems of escalating difficulty.

#### 90-Day Adherence to ART

We extracted pharmacy refill data for the preceding 3 months from pharmacy registers to calculate a measure of ART adherence, the medication possession ratio (MPR) [43]. The number of days that a participant had possession of antiretroviral medication in the 90 days prior to enrollment was divided by the number of days in the defined period (90 days), and multiplied by 100 to get the percentage; this value represented the proportion of days a participant had access to medication. The MPR has been shown to be a valid estimation of maximum possible adherence in settings such as Mozambique, where ART is provided for free through a single, centralized location, minimizing the possibility of individuals receiving medication from multiple source [5].

### Statistical Analysis

We report descriptive statistics as proportions, means with standard deviation (SD), or medians with interquartile range (IQR) as statistically appropriate. We evaluated internal reliability using the KR-20 [44]. We evaluated construct validity by examining the bivariate relationship between HIV-LT score and the seven factors selected a priori. We used the Spearman’s rank test to assess the correlation between the HIV-LT and continuous variables, including basic word reading, arithmetic computation, education, and 90-day adherence to ART. We used Kruskal–Wallis tests and Wilcoxon rank sum tests to compare the relationship between the HIV-LT and categorical variables including language spoken at home, occupation and income.

Using a split sample approach, we performed principal component factor analysis with an orthogonal rotation to determine the presence of multiple factors and item loadings on each factor. Additional exploratory factor analyses was performed including Principal Component Analysis, Maximum Likelihood Analysis, Principal Axis Factoring, General Least Squares Analysis with Orthogonal, Oblique and Equamax Rotations where applicable, to confirm the results of the original principal component analyses. We then created a shortened 10-item scale and tested for internal reliability by the KR-20. To establish construct validity of the HIV-LT10, we tested its association with the original 16-item HIV-LT and the seven variables selected a priori.

The National Committee of Bioethics for Health in Mozambique and the Institutional Review Board of Vanderbilt University reviewed and approved the protocol for this study. All study participants provided informed consent prior to enrollment in the study. We collected and managed data using REDCap^®^ electronic data capture tools hosted at Vanderbilt University [45]. We performed analyses using the STATA^®^ statistical software package v12 (College Park, TX, USA).

## Results

**Fig. 3:**
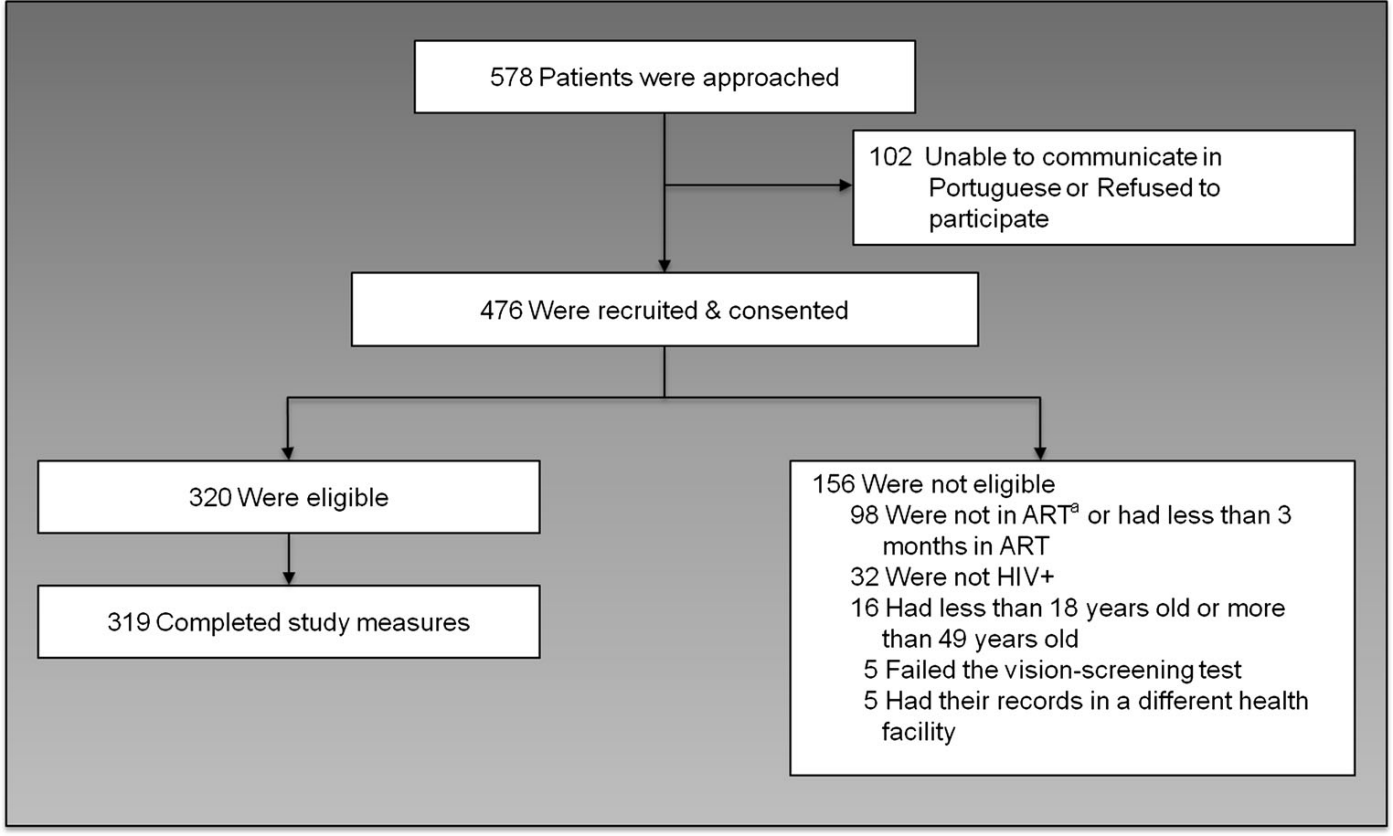
Study participant’s recruitment process. ^*a*^ *ART* antiretroviral therapy

We approached 578 potential participants from August to November 2012; 476 gave consent, 320 were eligible and 319 completed study measures. Lack of time and inability to communicate in Portuguese were the main reasons for non-participation (Fig. 3). The mean age of participants was 35 years (SD = 7), and most were women (76 %; Table 1). Most participants (71 %) lived within 10 km of the clinic. Only 49 % of the participants had a job outside the home, and most had a monthly income of <6000 meticais (US$200 in November 2012). Participants had a mean of 6 years (SD: 4) of formal education, and only 34 % used Portuguese as the primary language spoken at home. Participants had a median of 6 years since HIV diagnosis (IQR: 1–6) and median 2 years on ART (IQR: 1–4). Nearly two-thirds were in early stages of the disease (WHO stage I and II: 63 %). General literacy and numeracy median scores were skewed at 48 (IQR: 31, 54) of 57 for general literacy, and 25 (IQR: 21, 28) of 30 for general numeracy. Compared to participants in urban Polana Caniço, participants in rural Marracuene had lower incomes, less education, and lower levels of general literacy and numeracy. Fewer participants in Marracuene had jobs outside the home or used Portuguese as the main language spoken at home.

**Table 1:** Characteristics of 319 respondents attending HIV-related care at the urban Polana Caniço and rural Marracuene health centers in Maputo, Mozambique (August–November 2012)

Characteristic	Total (N = 319)	Polana Caniço (N = 181)	Marracuene (N = 138)	*P*
Age, mean (SD)	35 (7)	35 (7)	35 (6)	0.74
Female sex, %	76	76	75	0.94
Monthly income, %				
0–5999 meticais^a^	73	66	82	0.001
6000 meticais or more	27	34	18	
Job type, %				
Work outside the home	49	58	38	<0.001
Domestic or agriculture	23	11	41	
No job	27	31	21	
Distance from home to the clinic >10 km, %	29	29	30	0.84
Years of education, mean (SD)	6 (4)	7 (4)	5 (3)	<0.0001
Portuguese primary language at home, %	34	46	19	<0.001
Years since HIV diagnosis, median (IQR)	6 (1–6)	4 (1–6)	3 (1–5)	0.08
Years on ART, median (IQR)	2 (1–4)	2 (1–5)	2 (1–4)	0.02
HIV disease stage, %				
I	37	38	37	<0.001
II	26	18	37	
III	29	37	19	
IV	8	8	8	
90-Day adherence to ART (MPR), %	97 (92–99)	96 (87–98)	98 (94–100)	0.02
General literacy score,^b^ median (IQR)	48 (31, 54)	51 (41, 55)	43 (10, 52)	<0.001
General numeracy score,^c^ median (IQR)	25 (21, 28)	26 (22, 29)	24 (20, 28)	0.002
HIV-LT score, mean (SD)	42 (26)	48 (25)	33 (25)	<0.0001

*SD* standard deviation, *IQR* interquartile range, *ART* antiretroviral therapy, *MPR* medication possession ratio, *HIV-LT* HIV Literacy Test

^a^Monthly income in meticais, 0–5999 ≈ $0–$200 US dollars (November 2012)

^b^General literacy scores range from 0 to 57

^c^General numeracy scores range from 0 to 30

Mean score on the HIV-LT was 42 % (SD: 26 %, range 0–94 %). Participants in Marracuene had lower scores (mean: 33 %, SD: 25 %) than their peers in Polana Caniço (mean: 48 %, SD: 25 %) (*P* ≤ 0.0001). Table 2 displays the range of topics covered in the HIV-LT and how participants performed on each item. For example, 81 % of the participants were able to determine the amount of water needed to prepare an oral rehydration solution by reading instructions on a label. Only 57 % of participants were able to read the date of the next clinical visit in a commonly used appointment slip. Only 42 % of the patients were able to determine the number of pills to take each morning using written instructions, and only 46 % were able to tell how many pills would be needed for a 14-day trip if taking two pills a day. Even fewer (13 %) were able to interpret a commonly used prescription card with instructions for the correct time to take pills.

**Table 2:** HIV Literacy Test (HIV-LT) item content and correct responses among 319 adults on treatment for HIV infection

Question topic^a^	Correct, %
**1. Report the date of the next clinical visit presented in an appointment slip**	**57**
2. Determine the amount of water needed to prepare an oral rehydration solution by reading instructions on the label	81
3. Read a prescription card and, using the instructions, state the number of pills to take per day	66
4. Use a calendar to determine the date when a 30-day supply of pills will be completed	5
**5. Using a representation of a standardized dosing cup, indicate how to measure a dose of 7.5** **mL of zidovudine**	**48**
**6. Calculate the number of pills to take each morning using written instructions**	**42**
**7. Report the proper time to take a medication using written instructions**	**55**
**8. Interpret the value of CD4 count that would indicate the threshold for ART initiation**	**50**
**9. Interpret a dose of ART described as a fraction**	**43**
**10. Using a representation of a standardized dosing syringe, indicate how to measure a dose of 3** **mL of zidovudine**	**54**
**11. Calculate how many days a pill supply will last using written instructions**	**63**
**12. Calculate how many pills would be needed for a 14-day trip using written instructions**	**46**
**13. Interpret a prescription card with instruction for the recommended time to take pills**	**13**
14. Interpret the risk of mother-to-child transmission displayed in percentages	15
15. Understand the risk of opportunistic infection and malnutrition by calculating 10 % of a specific body weight	4
16. Understand the risk of treatment side effects when taking cotrimoxazole	29

^a^Bolded items correspond to items that were included in the 10-item version of the scale (HIV-LT10)

The HIV-LT had excellent internal consistency (KR-20 = 0.87) in this sample. As expected, higher HIV-LT scores were correlated strongly with higher general literacy (ρ = 0.8), higher general numeracy (ρ = 0.7) and more education (ρ = 0.7) (*P* < 0.001). Participants who worked in agriculture, had lower income, and did not use Portuguese as the primary language at home had significantly lower HIV-LT scores relative to their peers with higher socioeconomic status and/or who used Portuguese preferentially (Table 3).

**Table 3:** Association between selected respondent characteristics, HIV Literacy Test (HIV-LT) and the 10 question short HIV-LT version (HIV-LT10) scores among 319 Mozambican adults on antiretroviral therapy

Characteristic (N)	HIV-LT score		HIV-LT10 score^a^	
	Median (IQR) or correlation (ρ)	*P*	Median (IQR) or correlation (ρ)	*P*
General literacy score	0.8	<0.0001	0.7	<0.0001
General numeracy score	0.7	<0.0001	0.7	<0.0001
Education	0.7	<0.0001	0.7	<0.0001
Income		<0.0001		
0–5999 meticais (232)	38 (13–56)^b^		30 (10–50)^b^	0.0001
6000 meticais or more (87)	56 (38–69)^b^		60 (30–70)^b^	
Job				
No job (84)	50 (22–63)^b^	0.0001	40 (20–60)^b^	0.0042
Agriculture [51]	13 (6–38)^b^		10 (0–40)^b^	
Domestic (25)	44 (31–56)^b^		25 (10–50)^b^	
Work outside the home (155)	50 (25–69)^b^		50 (20–70)^b^	
Language spoken at home				
Portuguese (109)	63 (44–75)^b^	0.0001	60 (40–70)^b^	0.0001
Changane (170)	25 (13–50)^b^		20 (0–40)^b^	
Ronga (31)	50 (13–63)^b^		45 (10–60)^b^	
Other (9)^c^	31 (13–38)^b^		40 (15–60)^b^	
90-Day adherence to antiretroviral therapy	−0.01	0.79	0.001	0.99
Total HIV-LT score	Non applicable	Non applicable	0.96	<0.0001

^a^HIV-LT10 is the 10-item version of the HIV Literacy Test

^b^The HIV-LT and HIV-LT10 scores each range from 0 to 100 %

^c^Other languages: EChuabo and Chope

Median 90-day adherence to ART was high at 97 % (IQR: 91–99 %). HIV-LT scores did not correlate significantly with 90-day adherence to ART (ρ = −0.01, *P* = 0.8). Adherence to ART was similarly high for those participants with below average HIV-LT scores (median: 96 %; IQR: 84–98 %) and those with average or above average HIV-LT scores (median: 98 %; IQR: 92–99 %) (*P* = 0.7).

**Fig. 4:**
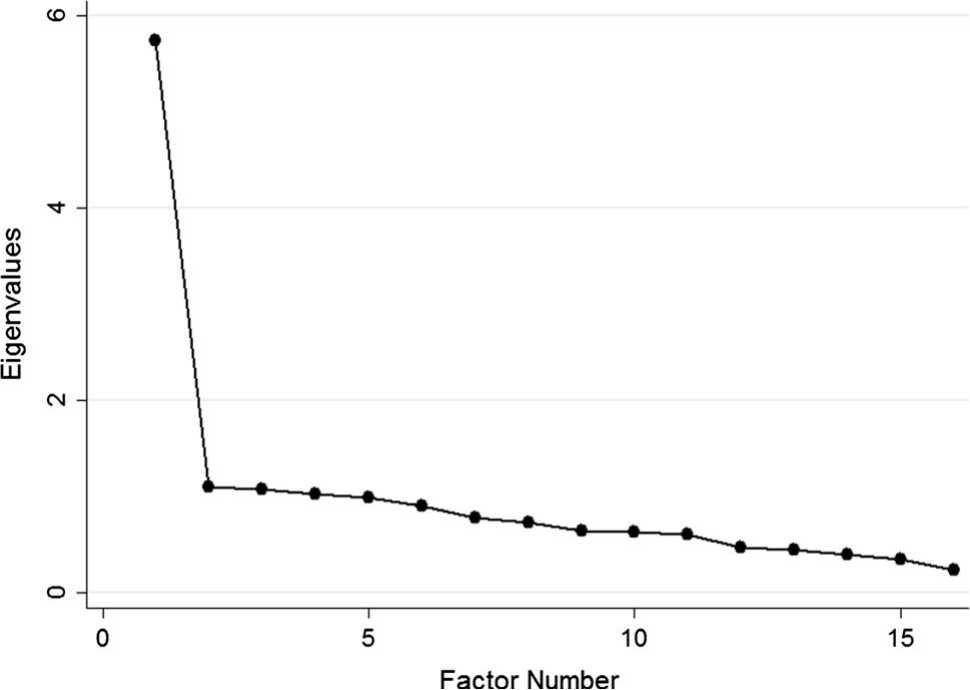
Scree plot of eigenvalues after Principal Component Analysis with orthogonal rotation

Principal component factor analysis showed that the 16-item HIV-LT loaded on one primary factor, as demonstrated by the significant drop in eigenvalues from factor 1 to factor 2 when compared to the other factors in the scree plot (Fig. 4). The additional exploratory factor analyses that was performed also suggested that there was only one factor underlying the 16 items in the HIV-LT. The shortened HIV-LT10 instrument had high internal reliability (KR-20 = 0.88), and we saw similar bivariate relationship with each factor used to evaluate construct validity as the 16-item scale (Table 3). Correlation between the HIV-LT10 and the original 16-item HIV-LT was very strong (ρ = 0.96, *P* < 0.0001).

## Discussion

The HIV-LT proved both reliable and valid in measuring health literacy skills among Portuguese-speaking Mozambican adults living with HIV, and represents a novel instrument that can be used to evaluate HIV health literacy. HIV-LT scores correlated in the expected direction with six out of seven variables selected a priori to test construct validity. No significant correlation between HIV-LT score and 90-day adherence to ART was found, which may have been due to participants’ surprisingly high level of adherence to ART (as measured by pharmacy refill data) in this study. The study highlights several potential challenges related to patient’s ability to understand daily HIV-related self-management tasks. Participants had difficulty interpreting commonly used prescription cards, understanding the correct time and number of pills to take daily, and understanding routinely discussed disease-related concepts, such as CD4+ T-lymphocyte cell count. The shortened 10-item version of the HIV-LT was also proven reliable and valid to measure literacy skills in the same population as the original scale. The HIV-LT10 is potentially more clinically useful, given time constraints of health care providers in low–and-middle income countries.

This study builds on the limited literature to date about health literacy and HIV in sub-Saharan Africa. We found only two other studies conducted in sub-Saharan Africa that have focused on measuring literacy’s role on HIV-related knowledge, behaviors and outcomes. One recent study reported an association between limited literacy and numeracy with lower HIV knowledge [19], and an observational study showed a relationship between higher self-reported literacy and virologic suppression of HIV [46]. Both studies applied suboptimal health literacy measures, since these were not specific to the disease or health context of the studies. The HIV-LT is one of a few examples of measures of health literacy that are specifically applied to HIV/AIDS [30, 32], and is the first to be developed within the sub-Saharan Africa context. Even in high-income countries, with a more substantial body of research on literacy and HIV, measurement of literacy in HIV-infected patients has been performed through the use of tools such as the Test of Functional Health Literacy in Adults (TOFHLA) [22, 34, 47, 48] and the Rapid Estimate of Adult Literacy in Medicine (REALM) [24, 28]. These tools still require patients to perform activities that are not specific to their disease or health condition, to apply quantitative information to a general health activities, or to simply read a set of health related terms. In contrast, the HIV-LT items include basic HIV-related treatment terms that are used routinely in care in Mozambique, and ask patients in treatment for HIV to demonstrate how they apply their range of literacy skills to daily self-management activities related to the disease. In a study conducted in South Africa, the REALM psychometric properties were tested and found inappropriate to measure literacy in the multi-linguistic South African population [17]. While some measures of health literacy have been translated and applied to non-English speaking populations [49], the settings were vastly different from the one found in sub-Saharan Africa. Hence, scales exhibit an inherent cultural bias that limits their use in non-Western populations. The HIV-LT items include culturally appropriate language and clinical materials used in daily care in Mozambique. This increases the scale’s face validity and may have resonated better with participating patients.

Our study did not yield evidence to support a relationship between health literacy skill and adherence to ART using pharmacy claim data. Our convenience sample of persons in HIV care and taking ART may have selected for more adherent persons. In fact, patients are initiated on ART once they demonstrate the potential for high adherence in Mozambique.

In higher income countries, data linking limited health literacy to lower adherence to ART have had inconsistent results [23, 26–28, 30–34, 50] and the relationship has not been studied previously in sub-Saharan Africa. Published studies have used a variety of methods to assess adherence to ART and health literacy, perhaps explaining the heterogeneity of results. Some studies report adherence to ART using patient recall, a skill that itself may be influenced by literacy [30, 31]. While pharmacy claim data are objective and straightforward to collect from routinely generated clinical data, they are limited by overestimation of actual pill taking if individuals discard or share pills and, therefore, can be considered to estimate maximum adherence, not likely adherence [51]. On items related to medication administration on the HIV-LT, patients had difficulty interpreting written prescriptions, and errors in interpretation may lead to errors in self-administration of HIV medication that would not necessarily be captured by pharmacy claim data. Half our study participants were patients who had been on ART for at least 2 years. We only assessed their adherence in the last 3 months. These patients may have had problems with adherence early in the treatment, but improved with counseling measures that have been in place in most of the HIV clinics in Mozambique.

Other study limitations include the fact that our assessment of health literacy was made as part of a hypothetical situation that may not reflect patient’s actual self-management skills or impact HIV outcomes. The recruitment of a convenience sample of urban and rural patients introduces the possibility of selection bias; patients who were not actively in HIV care, who had not been initiated on ART or who declined to participate may have lower literacy skills than those who participated in the study. Thus, health literacy skills among patients might be even worse than we have concluded. Another bias towards more favorable results than would be seen in a representative sample is our exclusion of non-Portuguese speaking patients from this study. Portuguese is the only official language in Mozambique, the language taught in schools, and used in the healthcare setting. Since Mozambican individuals who speak Portuguese have higher levels of education than non-speakers [19], they likely have higher literacy skill, and thus again their overrepresentation is likely to underestimate the magnitude of the relationship between health literacy and HIV-related activities reported in this study. The WRAT-3 scale, which we used to evaluate general literacy skills, is at best a measure of letter and word recognition, and focus primarily on arithmetic computation. However, studies by the WRAT developers and by our group have shown strong correlation between performance on it and other literacy, health literacy, and numeracy skills in other populations [52–56]. On the other hand, the scale was only a part of the construct validity model used and represents one of the most compelling reasons for the need for development of the HIV-LT—a better tool to assess a broader set of HIV related health literacy skills.

Identifying those with limited HIV health literacy is the first step in development of better strategies to communicate health information in a patient centered manner. In the US, an intervention tailored to improve communication of HIV-related information by using pictograph-guided adherence counseling to patients with limited literacy improved ART adherence and undetectable HIV viral loads, compared to patients submitted to general health counseling [57]. Improving health education materials, simplifying commonly used prescription cards and treatment instructions, or applying simple communication strategies such as the “teach-back/feedback model” are a few examples of interventions that can be tested and evaluated using the HIV-LT10 in future studies [48, 58–62].

## Conclusions

Health literacy skills are important to the daily management of HIV infection for Mozambican adults. The HIV-LT10 was designed to maintain items that assess HIV health literacy, and could more easily be used in the clinical setting to identify patients with limited health literacy skills who may benefit from tailored strategies to better communicate health information. The scale can also be used for future research focused in understanding the role of health literacy as a mediator of HIV-related behaviors and outcomes. Attention to patients and providers health communication skills can help improve the quality of health care, particularly in settings where low literacy is common.

## Electronic supplementary material

Below is the link to the electronic supplementary material.

Supplementary material 1 (DOCX 1014 kb)
