# Supplementary material for: Measuring Health Literacy Among Adults with HIV Infection in Mozambique: Development and Validation of the HIV Literacy Test
Source: AIDS Behav. 2016 Mar 10;21(3):822–32. doi: 10.1007/s10461-016-1348-3 (PMC5306223; doi:10.1007/s10461-016-1348-3)
Supplement: Supplementary file 1 — Supplementary material 1 (DOCX 1014 kb) [file 10461_2016_1348_MOESM1_ESM.docx]

**HIV Health Literacy Test (HIV-LT 10)**

The HIV Literacy Test (HIV-LT 10) is a shortened, more clinically useful version of the HIV Health Literacy test (HIV-LT), and it is designed to assess HIV health literacy skills for Portuguese- speaking persons living with HIV infection in Mozambique. Items in the measure assess a respondent’s ability to apply a range of literacy and numeracy skills to health-related activities necessary to participate in HIV-related care, including: the ability to dose oral medications, manage appointments, estimate risk of HIV transmission, and understand treatment side effects. When appropriate, items make use of clinical materials used during routine HIV care in Mozambique. Items are arranged in order of difficulty, from simple (reading the date of the next clinic appointment) to complex (interpreting the risk of components of mother-to-child transmission displayed as fractions) and are scored as correct or incorrect. In order to ascertain HIV related literacy skills even for those participants with very limited literacy, the first five items are orally administered.

**HIV Health Literacy Test 10**

*The administrator is to follow these directions:*

- Introduce yourself and hand a copy of the HIV-LT 10 to the participant.
- Hand the participant a pencil and paper before starting the test.
- Ask the participant to write any calculations if needed on the scrap sheet.
- Ask the participant to provide his or her final answers as recommended in each question.

**Please, read carefully. Write your answer in the space provided for each question.**

1. Look at the appointment slip. What is the date of the next clinical visit?


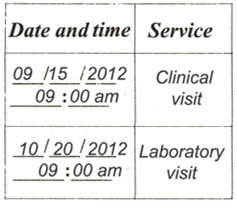


**ANSWER: ____________.** *(Correct answer: 09/15/2012 or 09/15; the Portuguese version will be changed to D/M/Y)*

2. Each morning, you take 2 pills of cotrimoxazole and 1 pill of duovir-N. How many pills do you take every morning?

**ANSWER: ____________.** *(Correct answer: 3 pills every morning; 3 pills; 3)*

3. You take 2 pills of your antiretroviral medicine every day. How many days will 10 pills last?

**ANSWER: ____________.** *(Correct answer: 5 days, 5; five)*

4. You take 2 pills to prevent Tuberculosis every day. How many pills would you need to take with you for a 14-day trip?

**ANSWER: ____________.** *(Correct answer: 28 pills; 28)*

5. Your doctor prescribed you an antiretroviral medicine. He told you to take 1 pill every 12 hours. You take the first pill at 8 am. What time should you take the second pill?

**ANSWER: ____________.** *(Correct answer: 8 pm; the Portuguese version will use a 24h clock)*

6. You should start antiretroviral treatment if your CD4 blood cell count is below 250 cells. Look at the boxes below that show CD4 blood cell counts. Circle the value of CD4 that would mean you should start treatment?


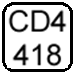

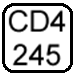

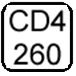


*(Correct answer: circle around the box with CD4 245)*


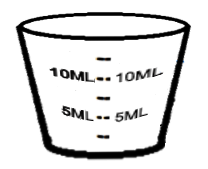
7. Your nurse gives you a syrup for your child. She tells you to give to your child 7.5 ml per day. On the picture below, color in with a pen where you would fill the cup with 7.5 ml of syrup?

*(Correct answer: mid-line between 5 mL and 10 mL)*


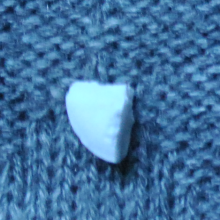
8. Your child takes ^1^/_2_ tablet of antiretroviral medicine in the morning. Look at the pictures of medicine below. Circle the picture that shows the correct dose of medicine to give to your child.


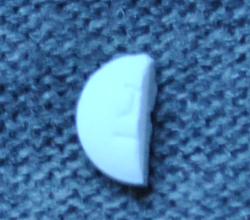

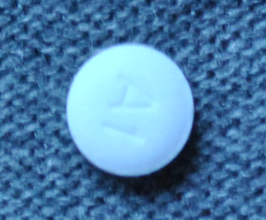


*(Correct answer: circle around the box with picture of half of pill)*


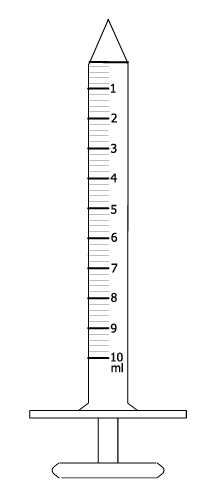
9. Your nurse gives you a syrup for your child. She tells you to give to your child 3 ml per day. On the picture below, color in with a pen, where you would fill the syringe with 3 ml of syrup?

*(Correct answer: line in front of*

*the number 3; anywhere between the 2.8 mL*

*and 3.2 mL is accepted)*

10. Look at the medicine card below. You take your breakfast at 7 am. What time would you take your morning pill?

**
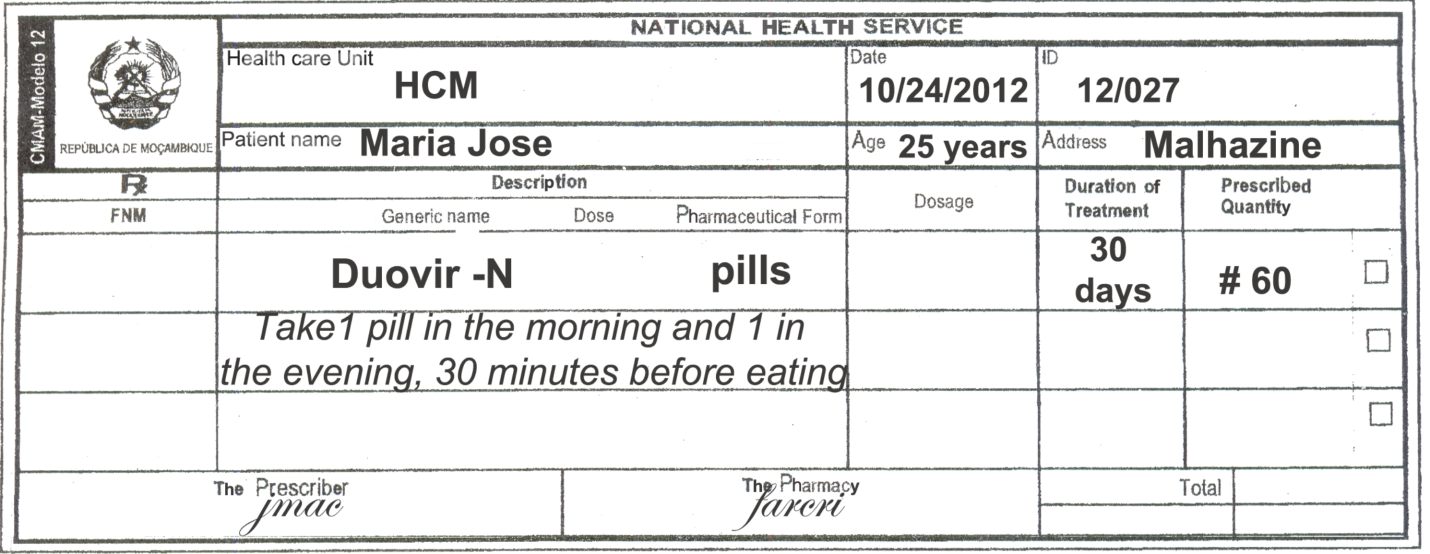
**

**ANSWER: ____________.** *(Correct answer: 6:30 am)*
